# Clothing Aesthetics: Consistent Colour Choices to Match Fair and Tanned Skin Tones

**DOI:** 10.1177/20416695211053361

**Published:** 2021-11-15

**Authors:** David Ian Perrett, Reiner Sprengelmeyer

**Affiliations:** School of Psychology & Neuroscience, 7486 University of St Andrews, UK

**Keywords:** clothing colour, fashion style, colour temperature, white skin tone

## Abstract

Fashion stylists advise clothing colours according to personal categories that depend on skin, hair and eye colour. These categories are not defined scientifically, and advised colours are inconsistent. Such caveats may explain the lack of formal tests of clothing colour aesthetics. We assessed whether observers preferred clothing colours that are linked to variation in melanin levels among White women. For this, we presented 12 women's faces: six with fair skin (relatively lower in melanin) and six with tanned skin (relatively higher in melanin). Across two experiments, observers (*N* = 96 and 75) selected the colour (hue and saturation or hue and value) of simulated clothing that most suited the skin tone of each face. Observers showed strong preferences for red and blue hues, and in addition favoured ‘cool’ blue hues to match fair skin and ‘warm’ orange/red hues to match tanned skin. This finding suggests that skin tone can determine colour preferences for clothes.

## Introduction

Clothing affects the perception of attractiveness and personality (e.g. Albright et al., 1988; Hesslinger et al., 2015). Colour is one aspect of apparel that may affect judgments, yet findings for the attractiveness of given clothing colours have been inconsistent. Initial reports that red enhanced attractiveness (e.g. Elliot & Niesta, 2008; Roberts et al., 2010) have been discredited (Francis, 2013; Hesslinger et al., 2015; Peperkoorn et al., 2016; though see Sidhu et al., 2021). It may not be that one size or colour fits all. Particular colours may look more appropriate on particular people. Fashion stylists’ advice is based on this assumption, yet there has been no systematic study of such advice. We aim to bridge that gap in knowledge by examining the perception of the aesthetic match between clothing colour and different people.

Apparel affects the wearer's self-confidence and esteem (Roberts et al., 2010), which contribute to general mental wellbeing (Mann et al., 2004; Sjögren-Rönkä et al., 2002). Approaching fashion aesthetics scientifically is important given these considerations and the monetary value of the fashion industry (∼$2.5 trillion, US Congress Joint Economic Committee Report, 2019).

We examine here whether there is consistency in public opinion about the aesthetics of colour matching between clothing and skin tone. There are extensive sources of stylist advice about clothes and skin colour but the advice is idiosyncratic and often obscure. Perhaps not surprisingly, the empirical testing of aesthetic choice of clothing colour is scant.

We first review general colour preferences, followed by contextual effects on colour preferences. We then focus on the stylist literature, attempting to glean rules for garment colour choice, and formulate predictions about garment colour preferences based on objective differences in skin complexion.

### General Colour Preferences

Several studies have observed preferences for green-blue hues (Palmer & Schloss, 2010; Palmer et al., 2013; Schloss et al., 2013; Westland & Shin, 2015) with blue often the favourite hue (Bakker et al., 2015; Dittmar, 2001; Eysenck, 1941; Schloss et al., 2013). Other studies have observed popularity of reds and blues and unpopularity of yellows (Guilford & Smith, 1959; Hurlbert & Ling, 2007; O'Donovan et al., 2011; Palmer & Schloss, 2010). Similar preferences have been found in other species; rhesus monkeys (Humphrey, 1972; Sahgal et al., 1975) and pigeons (Sahgal & Iversen, 1975) both prefer blue over other hues including yellow. These interspecies similarities suggest the possibility of an evolutionary dimension to colour preferences.

Palmer and Schloss (2010) argue that colour preferences derive from ‘emotional associations during a person's lifetime’. ‘The more enjoyment and positive affect an individual receives from experiences with objects of a given colour, the more the person will tend to like that colour’. Such emotional associations are likely to promote colour preferences that are largely context free (i.e. preferring the same colours for different objects). Hence, one expects similar colour preferences in most circumstances including fashion. The origins for the theory of colour associations underlying preferences can be traced back to the pioneer Fechner 1866 with his ‘Aesthetic Association Principle’ (Ortlieb et al., 2020).

### Context and Colour Preferences

Colour preferences can be influenced by colour–object associations (Strauss et al., 2013). For example, Palmer and Schloss (2010) presented participants with pictures of desirable red and undesirable green objects (e.g. strawberries vs mould) or, conversely, pictures of desirable green and undesirable red objects (e.g. trees vs blood). This viewing experience shifted colour preferences accordingly, showing that colour aesthetics can be modulated by valenced cues. The extent and duration of preference changes is not known.

**Figure 1.**
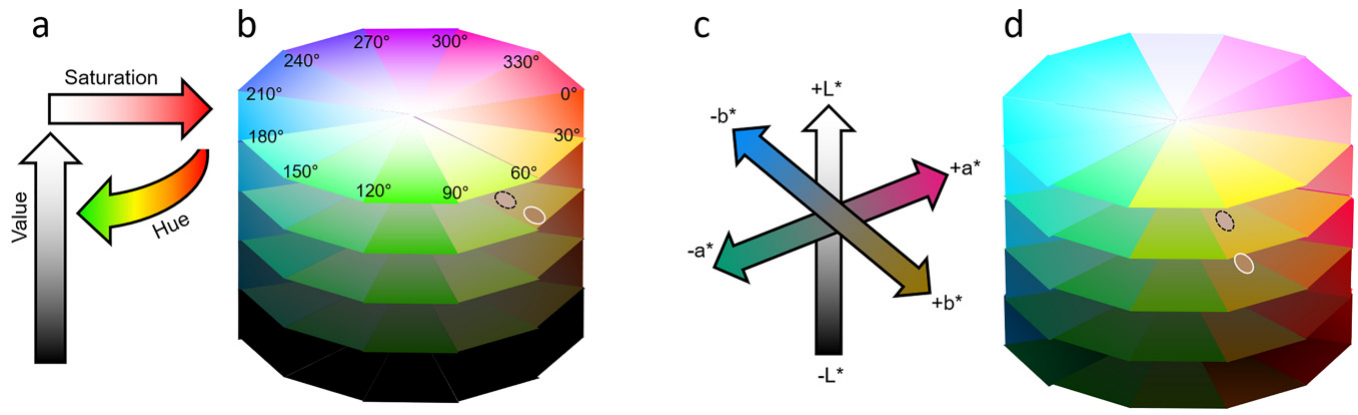
Colour spaces. ((a) and (b)) Depiction of colour space defined by hue, saturation and value (HSV, Tkalcic & Tasic, 2003). (a) HSV axes are illustrated as circular, horizontal and vertical arrows. (b) Five circular planes varying in hue and saturation. In each plane, hue is displayed in 12, 30° sectors. The vertically stacked planes are depicted for values of 0%, 25%, 50%, 75% and 100%. ((c) and (d)) Depiction of CIE L*a*b* colour space. (c) Three orthogonal arrows corresponding to the dark-light (L*), green-red (a*) and blue-yellow (b*) axes. The a* and b* axes are depicted at 50% lightness L*. (d) Five colour planes varying in a* b* are depicted at lightness levels of 0%, 25%, 50%, 75% and 100%. Each colour plane is constructed with 12 colour sectors matched to (b). Dashed black ellipse outline illustrates fair skin colour and white ellipse outline illustrates tanned skin colour. The differences in colour values for fair and tanned skin (see text for accurate values) are exaggerated by 100% for illustration and not intended to be realistic. ((a)–(d)) HSV and CIE L*a*b* colour values for the circular planes were defined using http://colorizer.org.

In other circumstances, it is less apparent that colour preferences depend on context. Early work indicated that different colours are preferred for different objects; for example, red is preferred for cars and blue for clothes (Holmes & Buchanan, 1984). Subsequent work concluded the opposite that colour preferences and meanings were largely similar across objects (e.g. Taft, 1997). Jonauskaite et al. (2016) used an optimization procedure to define most and least favoured colours. The selection of the colour of patches was made under three contexts: in general, for interior walls of a room or for a t-shirt. Most preferred hues were again red (hue angle 25^o^, see Figure 1 left) and blue-green (194^o^). These hues were the most preferred in general and for a t-shirt. Context had relatively mild effects on the most popular colours, yet the relatively unpopular yellow and orange were more preferred in the context of wall colours. For clothes, a person's colour preferences do not seem to differ from their general colour preferences (Bakker et al., 2015; Jonauskaite et al., 2016). Indeed, Lind (1993) reported that colours of favourite garments and dominant colours in the wardrobe were similar to stated general colour preferences.

Overall, this work indicates consistency in preferred colours and a small effect of the context of colour choice in low-valenced contexts (Jonauskaite et al., 2016). We therefore anticipate general preferences for blue-green and red colouring of clothes that will be independent of the person wearing the clothes.

### Colour in Fashion

One domain where there is extensive advice on colour aesthetics is that of clothing. Fashion stylists advise different clothing colours depending on a person's category based on eye, skin and hair colour.

The origins of stylist colour recommendations can be traced to Itten's classification of colours by contrasts (Bláha & Štěrba, 2014; Burton, 1984; Itten, 1973). Itten's categorizations included contrast in colour saturation (high vs low, Figure 1(a) and (b)) and contrast in warmth (Kalt–Warm–Kontrast or blue vs orange) (Bláha & Štěrba, 2014). The Kalt–Warm–Kontrast can split the circular array of hues (0–360°) into two halves, a ‘warm’ half centred on red or orange (approximately hue 360 or 0°) and a ‘cool’ half centred on blue (approximately hue 180°, Figure 1(a)). The binary application of Itten's contrasts of saturation and warmth allows colours to be allocated to four groups.

By analogy, the contrast of warm versus cold colour can be used to classify people's colouration into two categories (Burton, 1984). Additionally, the contrast of colour saturation has been used to allocate people to four categories, which are arbitrarily labelled Spring, Summer, Autumn and Winter seasons (Burton, 1984). This seasonal nomenclature was popularized by Jackson (1980) but there are alternative naming systems, for example, Star, Sun, Moon and Sunset (Hong & Kim, 2019).

Style advice suggests that people whose designated personal colour category is warm should wear colours that are also warm and that people whose designated category is cool should wear correspondingly cool colours (Burton, 1984). While the advice might appear intuitive, the classification of people into two or four categories is anything but clear. As noted by Yu et al. (2012): ‘the classification “guidelines” can be very obscure and cumbersome, arguably uninterpretable by a general user’.

Jackson (1980) describes people belonging to Winter and Summer categories as having a skin colour with ‘blue undertones’ (corresponding to cool colours), and those belonging to Autumn and Spring categories as having skin with ‘golden undertones’ (corresponding to warm colours). The methods for detecting the undertone colour of skin are subjective and sometimes circular. Circularity arises when the classification method is the same as the fashion advice itself (e.g. warm-coloured skin with golden undertones looks good with gold-coloured jewellery and warm clothing colours). For example, ‘Summers will improve their appearance in Summer's cool colours’ (Jackson, 1980).

A problem with the categorization is that multiple skin colours are allocated to one category. For example, Jackson (1980) describes Winters’ skin colour as being ‘gray-beige (ranging from light to dark), sallow or milky white’. Similarly, Summers’ skin can be ‘very fair, pale, but some have very pink skin, rose-beige (ranging from fair to deep rose-beige), sallow beige, and skin that tans’. Indeed, all four categories have a large range of skin colours. Classification is further complicated as the same skin colour name appears in multiple categories.

What is needed is objectivity in the allocation of categories. Computational approaches have used machine learning to train systems with example faces already categorized by stylists (Hong & Kim, 2019; Yu et al., 2012). The trained systems are then able to categorize new faces. Such automation does not make the classification rules explicit. Experts trained in one system may agree on recommendations but consistency does not imply validity.

### Stylists’ Clothing Colour Recommendations

Collin (1986) reported little consistency across 11 colour stylists’ colour recommendations and notes that there were more hue name recommendations for ‘Summer’ and ‘Winter’ in the green to purple (cool) segment of the colour circle than in the red to yellow (warm) segment. For ‘Autumn’ and ‘Spring’ the opposite was true, and most hue name recommendations occurred in the red to yellow segment of the colour circle (Collin, 1986). Thus, there is a vague recommendation for the matching of skin colour warmth to the colour warmth of clothes. This matching advice continues today: a survey of 20 websites for men and women's fashion (Appendix 1) shows the recommendation of warm-coloured clothes for a warm skin colour.

### Tests of Stylists’ Colour Recommendations

Francis and Evans (1988) selected eight garments (red and green in hue, high and low in brightness and high and low in feminine style). All the outfits were posed on a single model classified as ‘Spring’. The ratings of employability of the model did not offer support for stylist advice. Indeed, the authors conclude that clothing colour value (or lightness) but not hue is important for appearance judgments.

Radeloff (1990) compared the images of models dressed in clothing colours recommended and non-recommended for their seasonal category. While participants chose the recommended colour more frequently than chance, the test is inconclusive as a non-recommended colour (e.g. unsaturated yellow) could be unpopular for all models independent of their category. A definitive test needs to compare the same pair of clothing colours on different categories of model where one colour is recommended for one model but not for the second and *vice versa*.

Yu et al. (2012), Vogiatzis et al. (2012) and Hong and Kim (2019) have each designed automated systems for fashion style advice using the four ‘seasons’ colour scheme but no study has tested whether there is public agreement on the match between recommended colour of clothes and skin colour.

### Variation in White Skin Colour

While the allocation of people to one of four seasons is difficult to follow, it is possible that classification into warm and cold divisions using skin colour is more tractable. Differences in skin colouration are based on melanin, haemoglobin and carotenoid pigments. Melanin is perhaps the most obvious source of variation in skin tone. Melanin levels rise in response to sun exposure. This capacity to increase melanin levels (acquiring a suntan) is known as a facultative response but, even for body regions that are not exposed to UV, melanin levels differ between individuals due to genetic variation. Such variation reflects constitutive melanin levels. The facultative tanning response is closely linked to constitutive melanin levels. ‘Skin phototype I represents individuals with very fair skin who always burn and never tan when exposed to the sun’ while ‘phototype V represents individuals with very dark skin who rarely burn and tan profusely’ (Brenner & Hearing, 2008; Fitzpatrick, 1975, 1988).

### Colour Spaces

A colour space commonly used in computer graphics has three dimensions (hue, saturation and value or HSV, Figure 1(a) and (b)). Stylists’ colour recommendations are perhaps easiest to understand in terms of hue in the HSV colour space. An alternative colour space is that of the Commission Internationale de l'Éclairage (Figure 1 (c) and (d)), which is designed to be perceptually uniform so that colours separated by the same distance appear equally different. The CIE colour space is more suited to analysing skin colour and perception. The CIE space is defined by three orthogonal axes: dark-light (L*), green-red (a*) and blue-yellow (b*) (Figure 1(d)). The CIE colour axes reflect the colour opponency of human colour vision. Furthermore, skin pigments affect CIE colour axes differentially. Haemoglobin influences the green-red axis of skin colour; indeed, primate colour vision may have evolved to spot blood signalling in the skin (Changizi et al., 2006). By contrast, melanin affects the lightness of skin but also influences the blue-yellow axis of skin colour. For skin typical of White Europeans, higher levels of melanin are associated with a decrease in skin lightness and an increase in skin yellowness (Alaluf et al., 2002; Stephen et al., 2011). The decrease in lightness and increase in yellowness accompanying increased melanin levels are illustrated in Figure 1(c) by the positions of dashed and continuous ellipses.

Correlated colour temperature comes from the quality of light radiating from bodies at different temperatures and ranges from blue through yellow to orange and finally red. Hotter bodies at a higher physical temperature (12,000 K) emit a bluer light, while bodies at a lower temperature (2500 K) emit a yellow light, and those at the lowest temperature (1000 K) emit a red glow. The blue-yellow b* axis in CIE colour space relates to correlated colour temperature over the range of high temperatures (12,000–2500 K). The red-green CIE a* axis also relates to correlated colour temperature but over the lower range (2500–1000 K). Counterintuitively, ‘cool’ colours refer to blues, while yellows, oranges and reds are referred to as ‘warm’ colours.

Melanin differences may underly the stylists’ reference to warm and cold skin colour: warm being high in melanin and more yellow, cool being low in melanin and less yellow or bluer. Indeed, this link between tanned skin and a warm skin tone is explicit in current style websites; for example, ‘If you redden and burn easily and do not take on any “suntan” colour, you are cool toned. If you tan easily with very little to no burning, you are warmer toned’ (theglamgamer.com, 2020). Similarly Samotin (2020) states: ‘When you’re out in the sun, does your skin turn a golden-brown, or does it burn and turn pink first? If you fit into the former category, you’re warm-toned, while cool tones tend to burn (fair-skinned cool girls will simply burn)’.

### Our Testing

**Figure 2.**
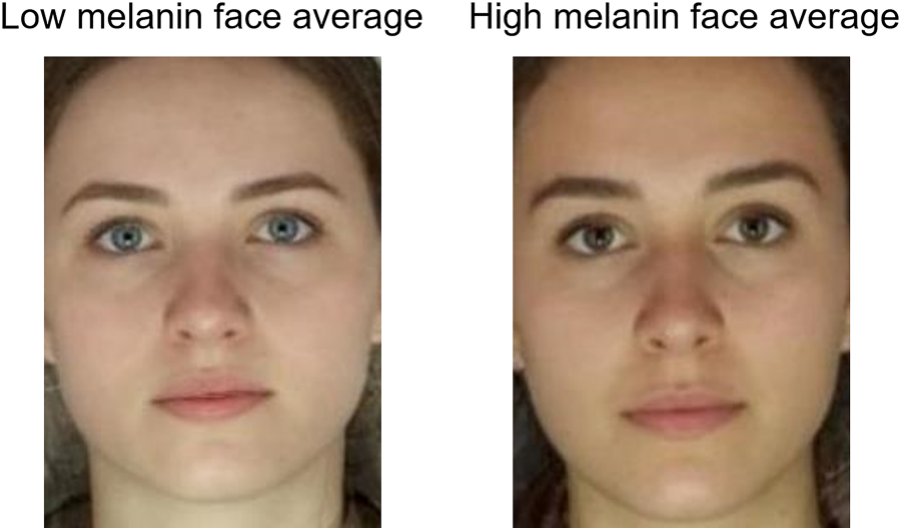
Average images illustrating differences in complexion between the fair and tanned faces. Average image of six women's faces with relatively low melanin pigmentation (fair skin, left) and average image of six women with relatively high melanin pigmentation (tanned skin, right). N.B. these (averaged) images were not part of the colour choice experiment but are included to illustrate the average differences in complexion between the two groups of face stimuli.

We base our experiments on different levels of melanin, testing stylists’ predictions for cool and warm skin colour (i.e. relatively low and high in melanin). For simplicity, we focus here on melanin level in the skin of young adult White women. Additional influences on clothing colour may derive from eye and hair colour (e.g. Jackson, 1980) but in general fair skin is associated with light-coloured hair and light (blue, green, hazel and grey) iris pigmentation (Han et al., 2008; Sturm & Frudakis, 2004; Sturm & Larsson, 2009). Darker, more tanned skin from higher levels of constitutive melanin in European populations is associated with dark hair and iris colour (see Figure 2). The associations reflect genes that are common to the control of melanin synthesis in the skin, iris and hair (Han et al., 2008; Sturm & Frudakis, 2004; Sturm & Larsson, 2009). Thus, recommendations based on hair and eye colour should be correlated with those based on skin colour.

If some colour (or colours) of clothing do look better on a particular type of skin, then the general public should show consensus and endorse that view by consistently selecting that colour (or colours). If the public do not show consensus, then there is no basis for stylists asserting that one colour or swatch of colours is advisable for those with a given skin colouration. This means, in principle, that we can use the opinion of a sample of the population to test stylist predictions of which colours go best with which skin colouration. Even in the absence of clear stylists’ predictions, one can still assess whether there are rules that the majority of participants follow in matching the colour of clothing to the skin colouration of the wearers.

Our aim is to assess consistency in colour choice for clothing in a sample of the public. With this aim in mind, we did not recruit expert stylists as participants. We note that the results from the public would have similar meaning independent of expert views. Indeed, if experts were to disagree with the public, then this would question the utility of expert advice.

### Hue, Saturation and Value

Colour preferences do not only reflect hue. Saturation (vividness, purity or chroma) and lightness (brightness or value, see Figure 1(a) and (b)) are additional determinants of preferences (e.g. Francis & Evans, 1988; Guilford & Smith, 1959; Hurlbert & Ling, 2007; Jonauskaite et al., 2016; McManus et al., 1981; Ou et al., 2004; Palmer & Schloss, 2010).

More vivid, saturated colours are preferred to desaturated colours (McManus et al., 1981; Palmer & Schloss, 2010), and lighter colours are preferred to darker colours (Camgöz et al., 2002; Guilford & Smith, 1959; McManus et al., 1981; Palmer & Schloss, 2010). Hence, all three colour HSV dimensions are linked to colour preferences and need to be explored.

It is not easy for observers to optimize colour when there are three dimensions to control. We therefore limited measures of observer preferences to two 2-dimensional arrays: one giving the full range of hues and saturation (while maintaining colour value at a constant maximum) and, reciprocally, a second array giving the full range of hues and value (while now maintaining colour saturation at a constant maximum).

### Hypotheses

We examine whether observers show consensus in colour choice for clothes, and whether the colour chosen for clothing is affected by skin colouration in White women. Specifically, we compare clothing colours chosen for relatively light or fair skin and relatively dark or tanned skin that are presumed to be relatively low and higher melanin levels, respectively.

We hypothesize (1) that colours chosen for simulated clothes will parallel general preferences for colours, showing popularity of blues and reds and aversion to yellow independent of the model wearing the clothes.

We hypothesize (2) that colours chosen for simulated clothes will differ in the context of skin melanin levels. In an interpretation of fashion stylists’ recommendations, we expect that clothing colours from the cool half of the colour circle (green, cyan, blue and violet, Figure 1 left) with hues 90–270° will be selected more frequently for fair skin than for tanned skin. Reciprocally, we expect clothing colours from the warm half of the circle (yellow, orange, red and purple) with hues 270–360° and 0–90° will be chosen more frequently for tanned skin. For completeness, we analyse colour preferences in relation to colour saturation and value of the HSV colour system.

Our study is exploratory and will examine preferred clothing colour defined by the CIE L*a*b* colour axes. One can interpret the stylists’ references to warm colours to predict that clothes with high values in the CIE a* (green-red) axis and high values in the CIE b* (blue-yellow) axis will be chosen for tanned skin tones. We are aware that the CIE b* blue-yellow colour axis is a major determinant of general colour preferences (Hurlbert & Ling, 2007). We therefore anticipate that this colour axis will be most important for choice of colour for clothes.

## Experiment 1

### Method

#### Stimuli

Forty-four participants, of which 35 were female and nine were male (aged 18–28 years with an average age of 21 years), were photographed in a neutral pose without make-up, under standard white illumination of three Verivide F20 T12/D65 daylight simulation bulbs in high-frequency fixtures (Verivide, UK) and frontal illumination using white projector lighting (6000 lumens NEC NP200). All participants gave permission for their image to be published. Six White female faces were chosen that were relatively dark skinned (high in melanin), and six White female face images were chosen that were relatively fair or light skinned (low in melanin).

To illustrate the difference in skin tone between the two sets of faces, Figure 2 shows the average image of the six faces with fair skin and the average image of the six faces with tanned skin. Procedures for averaging images are documented elsewhere (Tiddeman et al., 2001). Note that these averages were not used as experimental stimuli; experimentation was restricted to the original images of six fair and six tanned faces.

**Figure 3.**
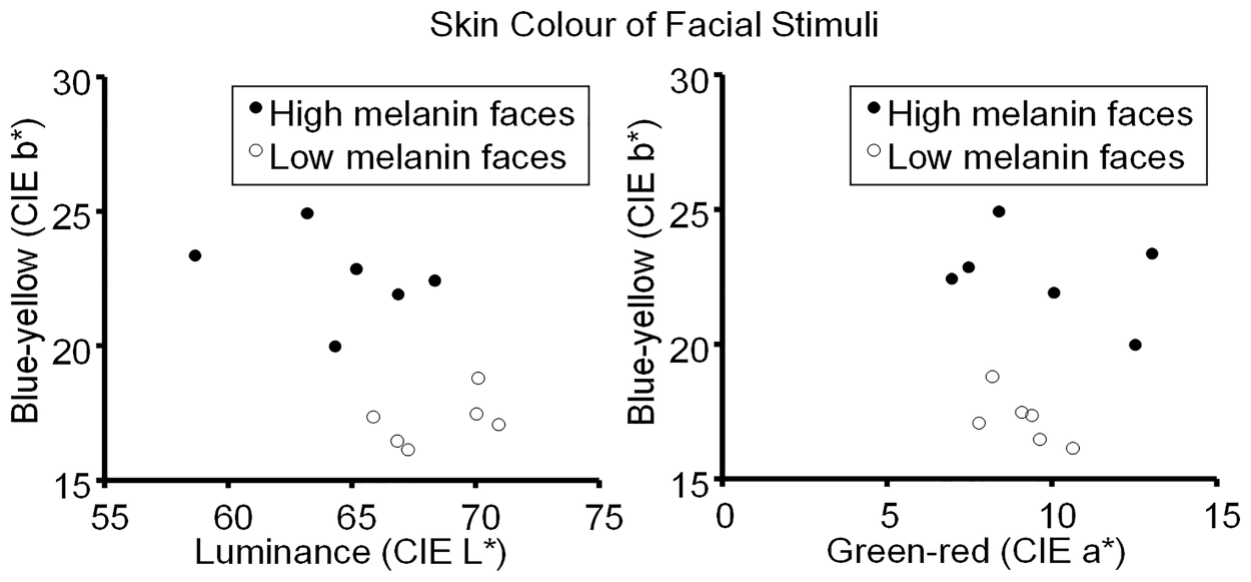
Scatter plots of the skin colour of the face stimuli. Left, CIE L* versus b* (lightness vs blue-yellow) values for the 12 face stimuli. Right, CIE a* versus b* (green-red vs blue-yellow). The plots show the non-overlapping range of skin tones for the six relatively low (open circles) and six relatively high melanin faces (filled circles).

The 12 images of the faces were aligned on their pupils, and rectangular image patches (free from hair) were cut from corresponding regions of the left and right cheeks and the forehead of each image. In each patch, the average colour of all the pixels was computed and the mean value across the three patches of the same individual was calculated. Comparison of the average colour of the image patches in CIE L*a*b* colour space confirmed that the two groups of face images were different in skin colour. As expected, the two groups differed in lightness L* (the fair-skinned group being lighter than the dark-skinned group: 68.51 + / − 1.92 and 64.43 + / − 3.08, mean + / − SD, respectively, *t* = 2.52, *df* = 10, *p* = .04, *d* = 0.85; Figure 3, left). Also as expected from differences in melanin, the two groups differed in the b* blue-yellow axis (the fair-skinned group having lower yellowness than the dark-skinned group: 17.21 + / − 0.85 and 22.57 + / − 1.50, respectively, *t* = −6.97, *df* = 10, *p* < .001, *d* = 2.46; Figure 3, left). The two groups did not differ in the green-red a* axis (fair-skinned 9.13 + / − 0.94, dark-skinned: 9.75 + / − 2.36, *t* = −0.55, *df* = 10, *p* = .60, *d* = 0.23; Figure 3 right). The skin colour differences between the groups were expected given the small sample sizes and because melanin has the largest effect on b*, a smaller effect on L* and no consistent effect on a* (Stephen et al., 2011).

The analysis in HSV colour space showed no change in hue (fair-skinned 26.15 + / − 1.79, dark-skinned: 27.67 + / − 3.22, *t* = −1.86, *df* = 10, *p* = .10, *d* = 0.60). Darker-skinned individuals were higher in colour saturation (fair-skinned 33.83 + / − 0.85, dark-skinned: 37.76 + / − 2.83, *t* = 5.92, *df* = 10, *p* < .001, *d* = 2.53) and showed a trend to be lower in colour value (fair-skinned 74.87 + / − 1.75, dark-skinned: 73.37 + / − 2.46, *t* = 2.25, *df* = 10, *p* = .051, *d* = 0.64). Thus the fair-skinned individuals are lighter and are lower in yellowness than the darker-skinned individuals as expected for differences in melanin but the two skin types do not differ in colour hue (see Figure 3).

Eye colour that also reflects melanin pigmentation was measured in similar fashion from square image patches centred on the left and right eyes. These patches showed that the iris colour was lighter in the fair than in the tanned skin faces (L* = 38.43 + / − 0.96, 28.11 + / − 2.15, respectively, *t* = 4.00, *df* = 10, *p* = .003, *d* = 6.19).

**Figure 4.**
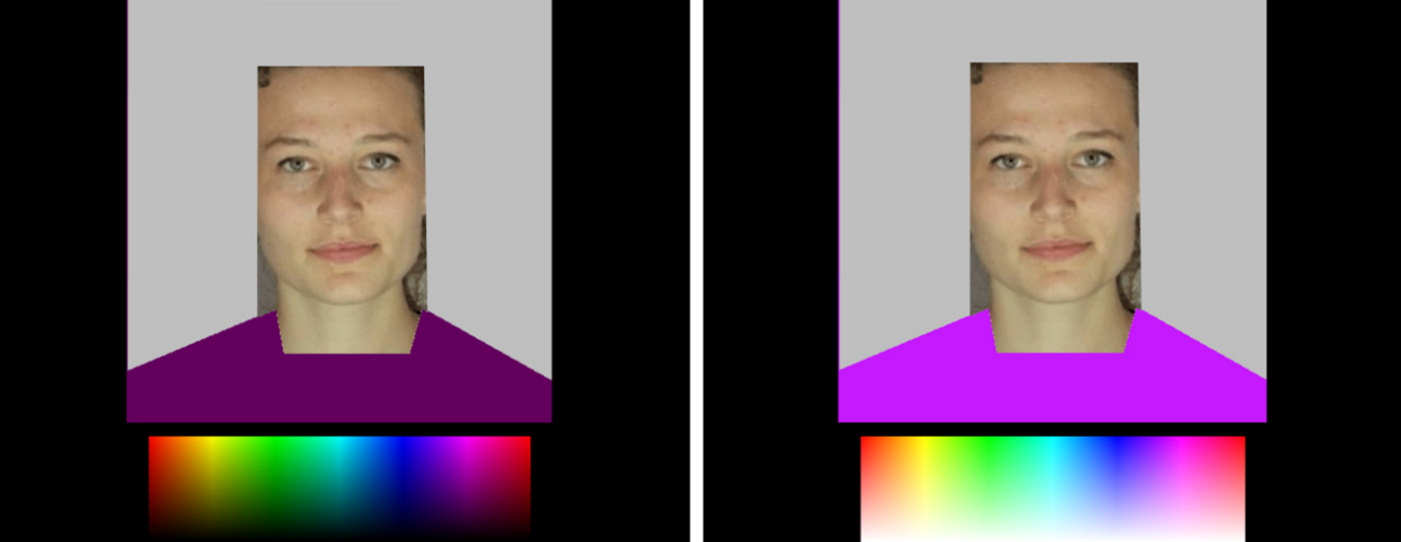
Illustration of stimulus interface for altering the colour of simulated clothing. The participant consented to their image being published. Left shows the interface for altering hue and value (saturation of colours fixed at 1.0), and right shows the interface for altering hue and saturation (value of colours fixed at 1.0). Participants picked any colour from the rectangular rainbow to fill the solid colour area of simulated clothing below the face.

Images were masked in PowerPoint with a light-grey (red, green, blue, RGB = 191/255, 191/255, 191/255) border to obscure most of the hair (see Figure 4). The images were then processed with Adobe® Photoshop®. In this program, matte white was overlaid to occlude the original clothes and adjusted in shape to simulate clothing. This white area was then converted to an alpha or transparent layer. Images were converted to GIF format with the transparent area saved as a second layer. Colour pickers were used to manipulate the second layer colour of the simulated clothing (e.g. Figure 4).

#### Participants

All participants gave their informed consent and ethical approval (Code PS13155) for the experiment was granted by the University of St Andrews, School of Psychology & Neuroscience Ethics Committee. One hundred participants were recruited from Prolific.co, an online participant recruiting platform. Their Prolific approval rating was  > = 83%, and all participants had a minimum of 10 Prolific submissions. We consider these measures as a proxy for seriousness to engage in this study. All participants were of UK nationality and had normal or corrected vision without colour anomalies. All participants completed the experimental task and a questionnaire which asked for self-report of ethnic group and colour vision status (‘As far as you know do you have normal colour vision? Please select: No, I have anomalous colour vision/ I am ‘colour blind’; Yes, I have full colour vision; Do not know; Prefer not to say’). Two participants reported an absence of full colour vision, one participant provided no answer, and one participant was unsure. This left a sample of 96 participants (gender: 65 female, 29 male, two did not report gender; age: mean + / − SD 25.5 + / − 4.6; range 18–35 years). Ethnicity reported was White (*n* = 79), Indian (South Asian, *n* = 7), African (*n* = 2), East Asian (*n* = 2), mixed ethnicity (*n* = 2) and other (*n* = 4).

#### Procedure

Participants were remunerated at a rate of £7.50 per hour. In each trial of the experimental task, participants were shown one of 12 female faces (six fair and six tanned skin) on their own computer screen and asked to adjust the colour of simulated clothing by using the cursor. Participants could pick any colour displayed in a rectangular rainbow. Trials were self-paced and typically took 15 s. Faces were presented approximately 3° × 1.5° in size and at a resolution of 300 × 150 pixels. The colour picker was approximately 4° × 1.2° and had a resolution of 400 × 120 pixels.

In one block of the experimental task with 12 trials, hue (0–360°) changed horizontally and value (0–1.0) changed vertically (Figure 4, left). In this block, saturation was fixed at 1.0. In the other block of the experimental task, also with 12 trials, hue (0–360°) changed horizontally and saturation (0–1.0) changed vertically. In this block, value was fixed at 1.0 (Figure 4, right). The 12 faces were presented in one block of trials with hue and value to be adjusted and in another block with hue and saturation to be adjusted. The uppermost pixel row of the colour control rectangles displayed the same range of hues at maximum saturation and value across both trial blocks. Block order was counterbalanced across participants, and the 12 faces were presented in random order within each of the two blocks.

Instructions were given prior to a block of trials, ‘Please adjust the colour of the clothing using the rainbow colour block’. On each trial participants were reminded ‘Move left-right and up-down to alter the clothing colour and brightness/saturation. Please change the colour of the simulated clothing so that it most suits the skin tone of the face. Please explore the full range of colour and brightness (or saturation)’ (see Figure 4). The initial colour displayed on each trial was set randomly from the full gamut of colours possible on that trial.

#### Analysis

To investigate the relationship between skin tone and colour of clothing we made a binary split of the colour circle (in HSV colour space) with cool hues defined as ranging from 90° to 270° and warm hues occupying the remainder of the circle (270–360° and 0–90°). For each subject, we computed the proportion of times cool hues were selected for clothing appropriate for faces with fair skin, and separately for relatively tanned skin colouration. These proportions were compared in a within-subject design. Standard deviations of means are given in text and standard errors in Figures. Data are available at https://doi.org/10.6084/m9.figshare.14596905.v1.

To examine clothing colour in terms of CIE colour space, we compared the average values on the L*, a* and b* (light-dark, green-red and blue-yellow) axes chosen for the six fair-skinned faces to the average chosen for the six tanned-skin faces in a within-subject design (matched-pairs *t* test).

### Results

#### Cold/Warm Colour Contrast and Clothing Choice

**Figure 5.**
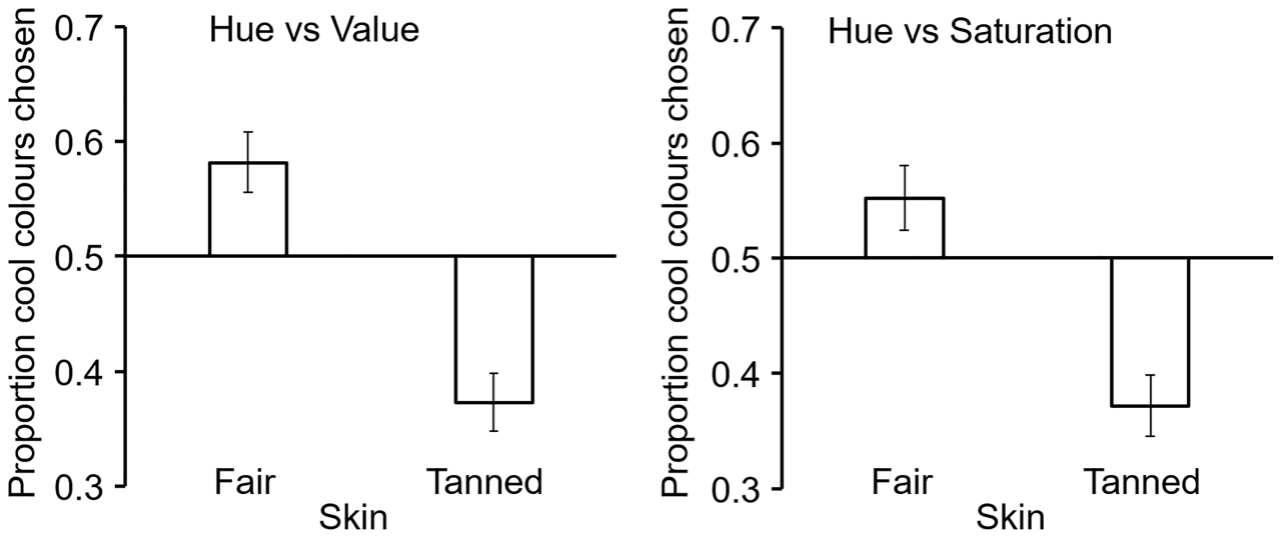
Illustration of clothing colour choice. Mean ( + / − SE) proportion of six trials on which cool colours were chosen for simulated clothing. Choices involved 96 participants each with six fair and six tanned faces. Trials altering hue and value (left) and trials altering hue and saturation (right). In both types of adjustment, participants were more likely to pick cool hues (90–270°) for simulated clothing when the stimulus face was fair skinned than when the stimulus face had tanned skin.

When adjusting the hue and value of simulated clothing, participants chose cool hues (90–270°) for fair-skinned faces more than they chose these hues for tanned-skin faces (fair skin: 0.58 + / − 0.26, mean proportion of trials cool colours chosen + / − SD, tanned skin: 0.37 + / − 0.25; *t* = 6.65, *df* = 95, *p* < .001, *d* = 0.68, Figure 5 left). Likewise when adjusting the hue and saturation of simulated clothing, participants chose cool hues (90–270°) for fair-skinned faces more than they chose these hues for faces with tanned skin (fair skin: 0.55 + / − 0.27, tanned skin: 0.37 + / − 0.26; *t* = 5.24, *df* = 95, *p* < .001, *d* = 0.53, Figure 5 right).

#### Hue

**Figure 6.**
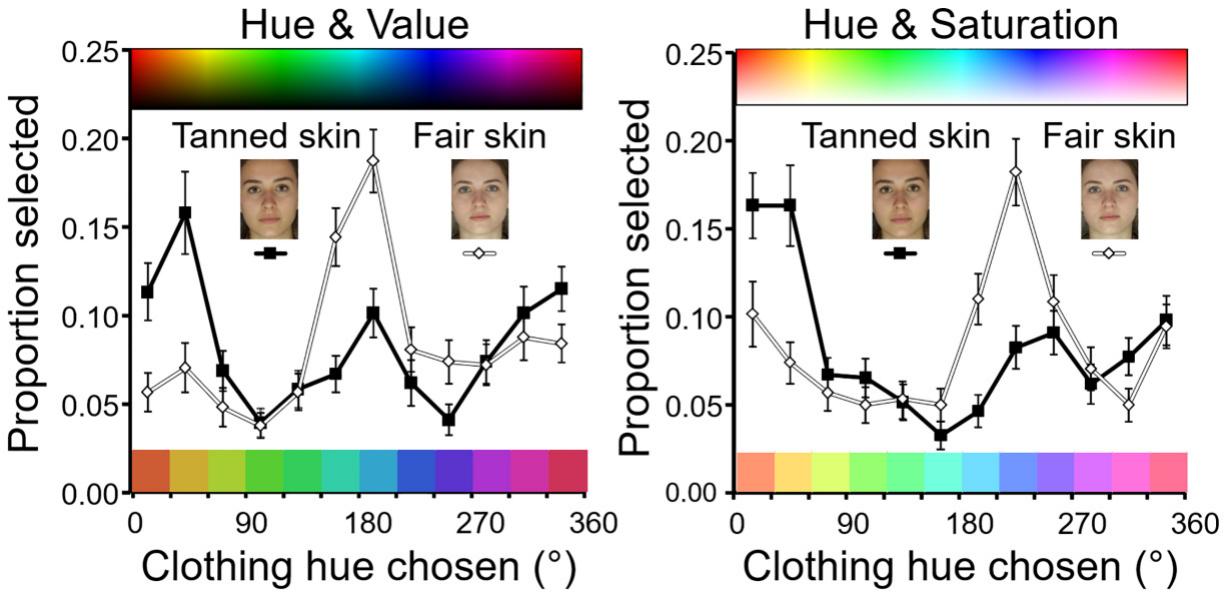
Clothing colour chosen. Left, participants selected hue (and value) from a rainbow rectangle like that shown at the top (see also Figure 4). Mean proportion of trials ( + / − SE) where 96 participants choose a given hue is plotted at 30° intervals across the hue range. The 12 colour patches at the figure base give the central hue for each 30° interval at the average value chosen by participants across the hue range. Solid (square) symbols and lines represent the average choice for the six women's faces with tanned skin. Open (diamond) symbols and lines represent the average clothing colour chosen for the six women's faces with fair skin. Right is a similarly constructed figure for hue versus saturation trials. Participants selected clothing colour from a rectangle varying in hue and saturation like that shown at the top. The 12 colour patches at the figure base present the central hue for each 30° interval at the average saturation chosen by participants across the hue range.

The binary split of hues into cool 90–270° and other warm hues may hide details of hue popularity. Therefore, a finer grain analysis was performed examining the impact of skin tone on colour choice in 30° hue bands across the hue range (see Figure 6).

Inspecting Figure 6 left and right, it is evident that there are popular hues in the orange-red and in the blue region of the colour circle (15–45° and 165–225°, respectively). Conversely, there are unpopular hues. For example, green (hue 105°) is less popular than blue or red across skin types. Hence, the popularity of different hues is somewhat independent of skin colouration yet there are marked popularity biases that do depend on skin colouration. Orange-red hues are more popular for tanned skin, and the blue hues are more popular for fair skin.

The method of testing also affects the popularity of the hue. When hue and value vary, then the most popular hue for fair-skinned faces is blue-green (195°, Figure 6 left) but when hue and saturation vary then the most popular hue shifts to blue (225°, Figure 6 right). The shift in hue selected occurs for both fair skin and tanned skin, although the shift is most evident for pale skin.

#### Saturation and Value

The average saturation of the simulated clothes chosen was lower for fair skin than for tanned skin (fair skin: 0.54 + / − 0.18, tanned skin: 0.59 + / − 0.16, *t* = −3.32, *df* = 95, *p* = .001, *d* = 0.34).

The average value of the simulated clothes chosen was not different for the two skin types (fair skin: 0.57 + / − 0.16, tanned skin: 0.55 + / − 0.17; *t* = 1.66, *df* = 95, *p* = .10, *d* = 0.17). The absence of a difference in selected colour value is salient because the skin lightness or value of the two groups of faces was chosen to differ.

#### Lightness, Redness and Yellowness (CIE L*,a*,b*)

We analysed the same data using the CIE L*a*b* colour coordinates. For the hue versus value trials, the average lightness (L*) of the simulated clothes chosen showed a trend to be higher for fair skin (39.63 + / − 14.53) compared to tanned skin (37.66 + / − 14.23; *t* = 1.87, *df* = 95, *p* = .07, *d* = 0.19). With the more constrained manipulation of lightness in the hue versus saturation trials there was no difference in the lightness selected for fair and tanned skin (75.01 + / − 10.95; 75.27 + / − 9.64; *t* = −0.26, *df* = 95, *p* = .80, *d* = 0.03).

When adjusting the hue and value of simulated clothing, participants chose a lower value on the CIE a* axis (more green, less red) for faces with fair skin (7.92 + / − 20.07) than for those with tanned skin (15.86 + / − 21.01; *t* = 6.65, *df* = 95, *p* < .001, *d* = 0.68). The preference for lower levels of CIE a* for fair-skinned faces was not significant when participants adjusted hue and saturation (fair skin: 14.67 + / − 22.97, tanned skin: 18.38 + / − 23.62; *t* = −1.36, *df* = 95, *p* = .18, *d* = 0.14).

**Figure 7.**
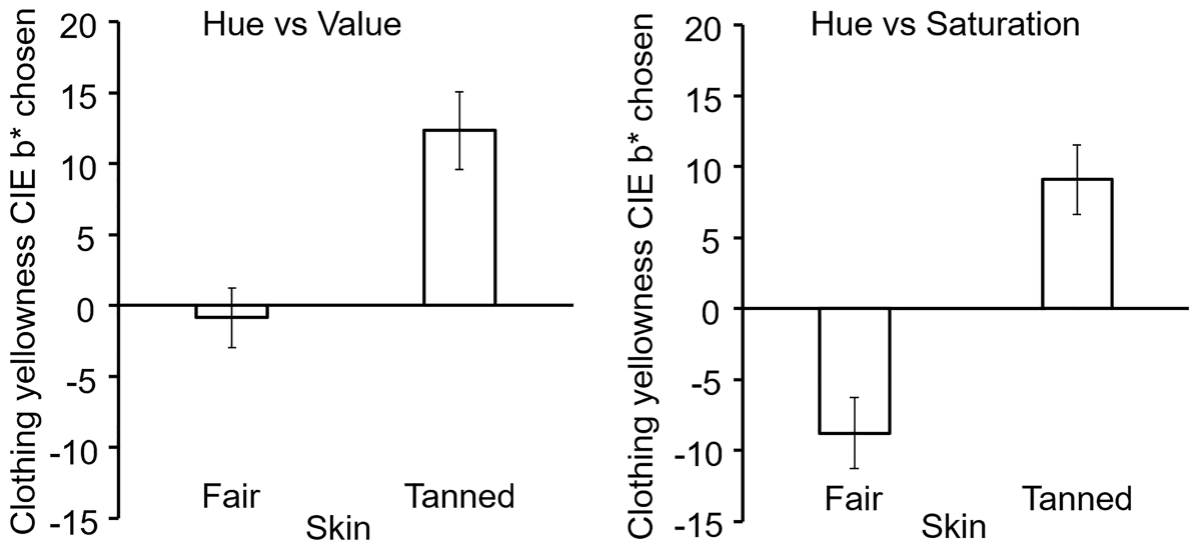
Clothing colour choice on the CIE b* axis. Mean ( + / − SE) of the CIE b* (blue-yellowness axis) chosen by participants. Left shows the results for trials involving adjustment of hue and value, and right shows the trials altering hue and saturation. In both types of adjustment, participants picked colours with higher b* levels for simulated clothing when the stimulus face was fair skinned than when the face had tanned skin.

When adjusting the hue and value of simulated garments participants chose a lower value on the CIE b* axis (more blue, less yellow) for clothes of fair-skinned faces compared to faces with tanned skin tones (fair: −0.86 + / − 20.94, tanned: 12.31 + / − 26.69; *t* = −4.57, *df* = 95, *p* < .001, *d* = 0.47, Figure 7 left). This preference for decreased levels of b* for fair-skinned faces was replicated when participants adjusted clothing using hue and saturation (fair: −8.80 + / − 24.84, tanned: 9.08 + / − 23.87; *t* = −5.44, *df* = 95, *p* < .001, *d* = 0.56, Figure 7 right).

#### By Participant Analysis

One can estimate colour bias in clothing colour choice from the proportion of stimulus faces where a bias is expressed (see Figure 5). One can also estimate colour choice bias from the percentage of participants who choose one colour range over another range. For the 96 participants, colour choice was averaged across trials varying in hue versus value and in hue versus saturation. Seventy percent (67/96) of participants were more likely to choose warm colour clothes for tanned than for fair skin. (16% (15/96) of participants did not express a preference.)

Across all trials, 65% (62/96) of participants thought clothes with higher CIE a* more appropriate for faces with a tanned skin compared to a fair skin tone. Across all trials, 77% (74/96) of participants thought clothing colour with a higher CIE b* content more appropriate for faces with a tanned skin compared to a fair skin tone. Hence, expressing colour in terms of CIE b* captures a colour dimension that matters for choice more than splitting the colour circle into warm-cold halves.

## Experiment 2

### Introduction

In Experiment 1, participants were instructed to change the clothing colour ‘so that it most suits the skin tone of the face’. The task does not necessarily measure preference or aesthetic judgment of clothing colour. Participants could have attempted to adjust clothing colour so that it matched the target face skin tone even if they did not like the colour chosen. We therefore reran a shorter version of the task with instructions changed to reflect an aesthetic judgment. The aim of our experiments was to establish whether there is consistency in public judgments of the aesthetic match between clothing colour and complexion of the wearer. We predicted that the results would follow those of the main experiment. We hypothesized that saturated colours with warm hues would be chosen for faces with a tanned complexion in preference to those with fair complexion.

### Method

#### Participants

Seventy-six participants were recruited from Prolific.co using the same screening criteria plus not having participated in Experiment 1. Data were excluded from one participant for not reporting normal colour vision. This left 75 participants (gender: 66 female, nine male; age: mean + / − SD 28.36 + / − 4.72; range 19–36 years). Ethnicity reported was White/Caucasian (*n* = 74) and other (*n* = 1). All participants gave their informed consent and the experiment was conducted under the ethical approval (Code PS13155) of the University of St Andrews, School of Psychology & Neuroscience Ethics Committee.

#### Procedure

The procedure was the same as Experiment 1 except that a subset of the original images was employed (four fair faces and four tanned faces). These images were presented in the hue versus saturation and hue versus value tasks. Each trial was accompanied by the instruction: ‘Alter the colour so that it looks most aesthetically pleasing for the person depicted’. Data are available at https://doi.org/10.6084/m9.figshare.14596905.v1.

### Results

In the hue versus value task, across the 75 participants the proportion of cool colours (hues 90–270) chosen was higher for the four fair-skinned (0.56 + / − 0.026, mean + / − SD) than for the four tanned-skinned faces (0.37 + / − 0.27, *t* = 4.33, *df* = 74, *p* < .0005, *d* = 0.67). In the hue versus saturation task, cool colours were again chosen more often for the fair (0.56 + / − .26, mean + / − SD) than for the tanned faces (0.37 + / − 0.27, *t* = 5.83, *df* = 74, *p* < .0005, *d* = 0.67). These results replicate the main findings of Experiment 1.

**Figure 8.**
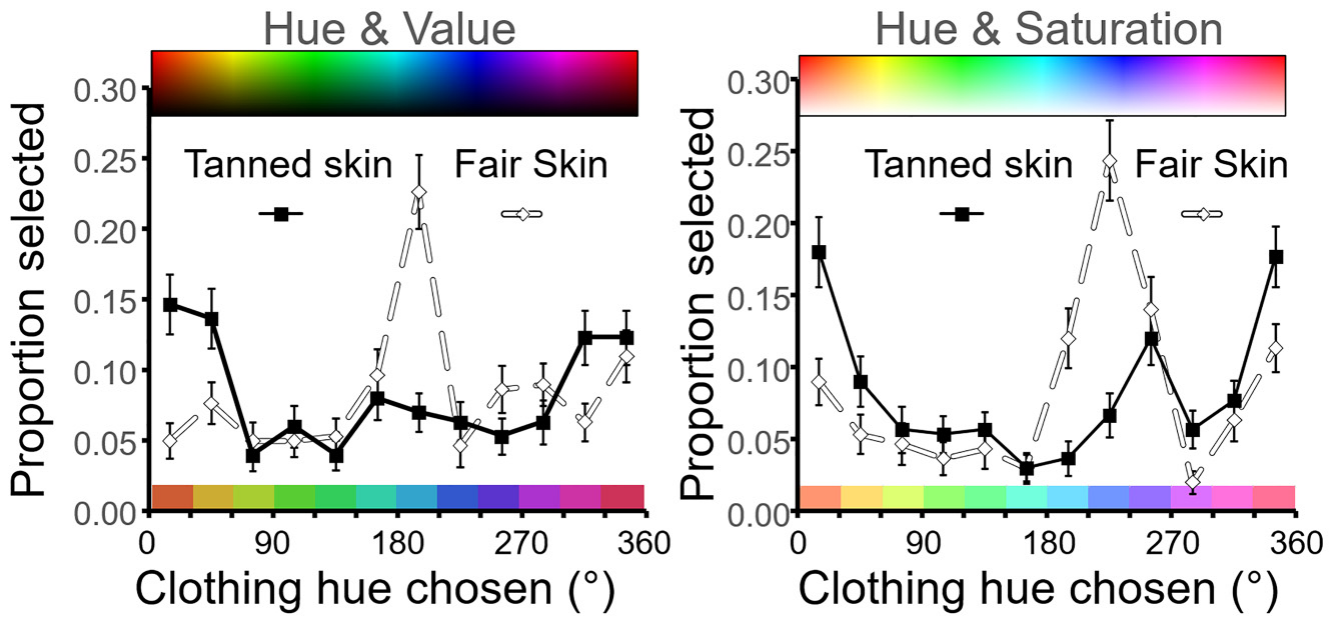
Clothing colour chosen in aesthetic judgments in Experiment 2. Left, participants selected hue (and value) from a rainbow rectangle like that shown at the top (see also Figure 4). Mean proportion of trials ( + / − SE) where 75 participants choose a given hue is plotted at 30° intervals across the hue range. Conventions are as Figure 6. Solid (square) symbols and lines represent the average choice for the four women's faces with tanned skin. Open (diamond) symbols and lines represent the average clothing colour chosen for four women's faces with fair skin. Right is a similarly constructed figure for hue versus saturation trials. Participants selected clothing colour from a rectangle varying in hue and saturation like that shown at the top. The 12 colour patches at the base of each figure present the central hue for each 30° interval at the average value or saturation chosen by participants across the hue range.

Figure 8 displays in more detail the impact of skin tone on colour choice across the hue range. In a similar fashion to Experiment 1 Figure 6, it is evident that orange-red (15–45°) and blue (165–225°) hues are popular whereas green (hue 105°) is unpopular independent of complexion. Hence, some hues are judged aesthetically appealing in general, yet aesthetic judgments are also contingent on complexion. Orange-red hues are judged more aesthetic for tanned skin and the blue hues more aesthetic for fair skin.

As Experiment 1, participants chose clothing colours with a slightly lower saturation for the four low-melanin faces (0.59 + / − 0.17, mean saturation + / − SD) compared to the four higher-melanin faces (0.61 + / − 0.17) but this difference did not reach significance (*t* = −1.41, *df* = 74, *p* = .161, *d* = 0.18). Participants chose clothing colour at similar lightness (value) for fair-skin (0.52 + / − 0.16, mean value + / − SD) and tanned-skin faces (0.51 + / − 0.17) (*t* = 0.62, *df* = 74, *p* = .539, *d* = 0.07).

Following Experiment 1, we estimated the proportion of participants with a bias in their colour choice. Seventy-one percent (53/75) of participants were more likely to choose warm colour clothes for tanned than for fair skin. (13% (10/75) of participants did not choose warm colours differently.) Seventy-three percent (55/75) thought clothing with a higher CIE b* content more appropriate for tanned than for fair skin. Hence, CIE b* is a colour dimension that accounts for choice just as well or slightly better than the warm-cold split of the colour circle.

### Discussion

#### Summary

Our results indicate consistency in participants’ colour preferences for clothes. Consistency refers to the similarity of results over two different tasks (hue vs value and hue vs saturation), instruction sets (matching the skin tone and aesthetically pleasing) as well as over different groups of participants and multiple faces. In support of our first hypothesis, we find two distinct popular hue ranges that lie almost opposite one another in the colour wheel, at hue angles 0–45° and 180–225°. The popularity of these red and blue colours for clothing tallies with previous studies showing the popularity of blue and red tones when general hue preferences are measured without context.

Additionally, we found that the preferred colour of clothing is context dependent. Clothes with blue hues are preferred for individuals with fair skin and clothes with yellow-red hues are preferred for individuals with tanned skin. While stylists’ recommendations for clothing colours are ambiguous (Collin, 1986; Yu et al., 2012), we found evidence support for our second hypothesis and the general maxim that clothes with a warm colour are more suited to individuals with a warm skin tone and, reciprocally, cool colour clothes more suited to individuals with a cool skin tone (Burton, 1984).

Figures 6 left and right shows that the preference biases are not for the entire warm or cool half of the colour circle but are more selective and reflect choices of hues 0–45° for fair skin and hues 180–225° for tanned skin. The preferences for these hues are evident in both the value and saturation tasks.

A modest improvement in describing preferences for clothing colour comes from focusing on a different dimension of colour. Compared to the colour contrast in terms of cold versus warm, the blue-yellow colour axis (CIE b*) accounts for a greater proportion of variance in preferences (see also Hurlbert & Ling, 2007). In Experiment 1, 77% of people chose clothes with higher b* colour for tanned skin, whereas 70% chose clothes from the warm half of the colour circle.

#### Advances of This Study

There are two domains where our methods offer advance. The first advance is in allocation of people to categories. Stylist advice on categorization is obscure (Collin, 1986; Yu et al., 2012). We base categories on a perceptually evident dimension (level of melanin), which can be confirmed with objective measures of skin colour in images.

The second contribution of our study is in defining clothing colours appropriate to skin-tone categories. We use a simple assessment of what a cross-section of the public think is a good colour of clothing to match skin tone. Others have used photometry (e.g. MacFarlane et al., 1994) or image analysis to segregate skin types (Hong & Kim, 2019; Yu et al., 2012) to derive garment recommendations but previous studies have not tested whether the public agrees or disagrees with the colour recommendations.

#### Colour Preference, Contrast and Assimilation

The colour choices of simulated clothes observed here could reflect the dynamic interaction of multiple influences.

The first influence would be the overall colour preference for red and blue hues that is typical of general colour preferences (Fortmann-Roe, 2013; Hurlbert & Ling, 2007; O'Donovan et al., 2011; Palmer & Schloss, 2010; Palmer et al., 2013; Schloss et al., 2013).

The second influence could be colour contrast (Stockman & Brainard, 2010) with the skin tone taking on an induced hue, complementary to the colour of the adjacent simulated clothes. Colour contrast from red clothes would induce an unhealthy appearance of skin lacking in oxygenated blood (Henderson et al., 2017). Participants would be expected to be biased away from red clothes particularly when the face has fair skin, since pale faces require the most reddening to look maximally healthy (Stephen et al., 2009a, 2009b). By contrast, tanned faces need less skin reddening to look their healthiest. This might allow a greater tolerance of red clothing for those with tanned skin. Hence for tanned faces, participants might express their general preference for red in their choice of colour clothing. The above explanation relies on colour contrast and general preferences. A full explanation, however, needs to account for the lack of popularity of blue clothing for tanned faces. Colour contrast with blue clothing would be expected to induce an increase in skin yellowness, which should be beneficial to all faces including those that have a tan (Stephen et al., 2009b, 2011). Admittedly, the appearance gain from a colour contrast with blue clothing will be highest with fair skin (Stephen et al., 2009b, 2011), which may explain the greater popularity of blue clothes for fair skin.

The third influence might be colour assimilation which has an effect that is opposite to colour contrast. Through colour assimilation one might expect red clothing colours to induce a red colour in the skin. Colour assimilation where the facial skin takes on the same colour as that of an inducing element has been noted. For example, red eyeshadow or red lipstick makes the face look redder and more attractive (Kiritani et al., 2016, 2017).

We acknowledge that the above explanations are not very satisfactory as they lack parsimony and involve two or three contributions.

#### Stereotypic Associations and Preferences

A different type of explanation is based on natural selection and stereotypic associations. Melanin has protective properties against UV damage from sunlight exposure, yet too much melanin disrupts UV-aided synthesis of Vitamin D and impairs dietary calcium absorption. These competing pressures explain the Polar-Equatorial gradient of melanin levels with melanin being raised in populations that historically lived closer to the Equator (Jablonski & Chaplin, 2000; López & Alonso, 2014) and were more exposed to UV radiation. Hence, natural selection has ensured that darker skin is associated with a warmer climate closer to the Equator and fair skin is associated with a cooler climate at higher latitudes.

We suggest that the Polar-Equatorial gradient in melanin underlies the clothing colour aesthetics observed. Our participants may be reflecting the geographical association of warmth and tanned skin when making their colour choices. A tanned skin high in melanin has connotations of a warm ambient temperature and may be matched to a garment colour which shares warm temperature associations. Indeed, in Experiment 1, the participants’ task was to choose the colour of clothing ‘so that it most suits the skin tone of the face’. This reliance on associations does not detract from the aesthetic appreciation of perceptual matches. Associations have been used to explain general blue-red colour preferences (Hurlbert & Ling, 2007; Strauss et al., 2013) and personality inferences from the colour of clothes (Pazda & Thorstenson, 2019). Indeed, familiarity has been suggested to encourage processing fluency and enhance aesthetic judgments in many perceptual domains (Reber et al., 2004). Our suggestion here is that familiar associations extend to aesthetics of colour matches between clothes and skin tone. This account of clothing colour preferences linked to associations between skin tone and temperature is, of course, post hoc and needs evaluation.

#### Lightness and Colour Saturation and Complementarity

While the two groups of stimulus faces differed in skin lightness, there was a trend for observers assigning higher lightness clothes only to fair skin. This lack of effect is surprising since lightness (or value) is important in colour preferences (Camgöz et al., 2002; Guilford & Smith, 1959; Jonauskaite et al., 2016; McManus et al., 1981; Palmer & Schloss, 2010). Our results do not deny the importance of lightness in garment aesthetics but indicate clothing lightness is not chosen to match or contrast with fair and tanned skin. The colour saturation of clothes chosen for faces with tanned skin was significantly higher than for those with fair skin in Experiment 1. Stylists’ recommendations are unclear with respect to colour saturation, so we made no specific hypotheses about saturation.

Deng et al. (2010) found that consumers chose colours matching in saturation when designing outfits from multiple components. This might suggest that observers prefer clothing and skin colour to be matched in saturation (as they would for two garments). It should be noted that the colour saturation selected for clothing was high (54% for fair and 59% for tanned skin) compared to the colour saturation of the facial skin (30% for fair and 38% for tanned skin). Thus, while the order of colour saturation was matched between faces and clothes, saturation was not matched in magnitude.

Clothing colour choice could be based on a selection of colours that are complementary to skin tones. Complementary colours lie on opposite sides of the circular array of hues (see Figure 1) and when mixed together produce an achromatic shade of grey. We note in the Methods that fair and tanned skin tones were very close in hue (fair-skinned 26° and dark-skinned 28°). Complimentary colours for all of the faces would be very similar blue hues (205° for fair and 208° for tanned skin). Thus, clothing choice does not seem to be based on colour complementarity in the technical sense.

#### Limitations

The current study has several limitations. We performed testing across the internet and hence different participants would have different colour viewing conditions. Nevertheless, our findings were robust in generalizing across this variation in viewing conditions. In other unpublished work using colour-calibrated presentation we find equivalent preferences of cool hues for fair-skinned faces and warm hues for darker-skinned faces. Our study faces were restricted to young adult White women. Further, the sample size of faces was small and was comprised of six with fair and six with tanned skin. We do not know if our findings will extend to faces more varied in age, gender and ethnic demographics. Future research should examine colour of clothing in relation to a full range of melanin levels defined using spectrophotometry and referring to the Fitzpatrick skin phototype scale (Astner & Anderson, 2004; Fitzpatrick, 1975, 1988).

We have worked only with light skinned faces. We note that for individuals with darker skin (CIE L*<50) the relationship between melanin level and skin yellowness reverses and increased melanin levels are associated with lower skin yellowness (Alaluf et al., 2002). The relationship between preferences for clothing colour and melanin levels in faces with dark skin (L*<50) could be an interesting testbed for the explanations of clothing aesthetics.

Our participants were also relatively homogenous, predominantly self-reporting as White, young adult in age and female in gender. There are differences in hue preferences according to culture and gender (e.g. Al-Rasheed, 2015; Sorokowski et al., 2014), age and education (Bakker et al., 2015; Dittmar, 2001). Hence, a broader participant demography could qualify the clothing colour preferences reported here. Fashion preferences, including colour, change over time (Frassanito & Pettorini, 2008). It will therefore be important to test whether the associations of skin tone with clothing colour are durable or a passing fashion.

Our findings appear to reflect levels of skin melanin. Of course, there are other dimensions of skin colour reflecting levels of blood and carotenoid pigments and the distribution of melanin within the skin. Further work could establish whether these alternative skin colour dimensions (or age and make-up) influence choice of clothing colour.

Our simulated clothes were flat colour shapes. An increase in the realism of clothes is desirable in future work.

### Conclusion

Our research establishes for the first time that there are rules relating the aesthetics of garment colour to objectively measured skin tone. In accordance with our Hypothesis 2, most of the population studied (77% in Experiment 1) shared the opinion that a warmer clothing colour with a higher b* value is more suitable for women with tanned skin than women with a fair skin tone. In short, blue hues were chosen for fair skin and orange-red hues were chosen for tanned skin. While stylists’ advice remains obscure, inconsistent and pricey, we show that it is possible to build an evidence base from which validated style advice can be generated and given freely. In a similar manner to studies of facial attractiveness, we find that it is possible to build a science of aesthetics for clothing that is based not on self-appointed expertise but on preferences of the majority of the public.

## Appendix 1

Fashion sites giving advice about cool and warm skin types

https://www.wikihow.com/Choose-Colors-That-Flatter-Skin-Tone

http://www.instyle.co.uk/how-tos/how-to-find-best-color-to-wear-for-your-skin-tone

http://www.thelist.com/20621/best-clothing-colors-skin-tone/

https://www.realsimple.com/beauty-fashion/clothing/shopping-guide/what-colors-look-best-me

https://verilymag.com/2015/10/color-theory-which-colors-suit-your-skin-tone-style

http://stylecaster.com/cool-warm-skin-undertones/

https://www.colorpsychology.org/the-best-clothing-colors-for-different-skin-tones/

http://womens-fashion.lovetoknow.com/What_Colors_Look_Good_on_Me

https://blog.stitchfix.com/fashion-tips/warm-and-cool-how-to-dress-for-your-skin-tone/

https://www.colormepretty.co/categories-2/4-season-color-analysis/

https://artofstyle.club/the-right-colors-for-your-skin-tone/

http://attireclub.org/2013/04/18/choosing-the-colors-of-your-clothes-according-to-your-

https://www.thedarkknot.com/blogs/suitupdressup/how-to-dress-for-your-skin-tone-mens-skin-tone-style

https://bespokeunit.com/color/skin-tone/

http://sexystyleforjoe.com/how-to-wear-colors/

https://www.gq.com/story/how-to-know-what-colors-look-good-on-you

https://restartyourstyle.com/776/warning-wearing-color-can-make-you-look-like-crap/

https://2ndmostinterestingwoman.wordpress.com/2015/12/27/color-analysis-for-men/

https://woodnax.com/blogs/news/139696199-your-perfect-colors-the-right-colors-for-your-skin-tone

http://everyguyed.com/fashion-101/how-to-choose-clothing-colors-that-flatter-your-skintone/
